# Mr. John Edward Platt

**Published:** 1910-08-20

**Authors:** 


The death is announced at Manchester of Mr. John Edward Platt, who was forty-four years of age, and for some years chairman of the medical board, member of the board of management, and honorary surgeon to the Manchester Royal Infirmary, lecturer on practical surgery at the University of Manchester, honorary consulting surgeon to the local hospital for consumption and the Warehousemen's and Clerks' Orphan Schools, major of the 2nd West General Hospital (Territorial), and formerly surgical officer at the Manchester Cancer Hospital, and resident medical officer at the Barnes Convalescent Hospital, Cheadle. Mr. Platt was joint editor of the Manchester *Medical Chronicle* and a voluminous contributor to surgical literature. He was a Manchester student, but qualified M.B., B.S. London. He later on obtained the F.R.C.S. Eng., and the M.S. London, and was awarded several gold medals and prizes.

